# Genotypic distribution and hepatic fibrosis among HIV/HCV co-infected individuals in Southern China: a retrospective cross-sectional study

**DOI:** 10.1186/s12879-015-1135-1

**Published:** 2015-09-30

**Authors:** Kali Zhou, Fengyu Hu, Charles Wang, Min Xu, Yun Lan, Jamie P. Morano, Stanley M. Lemon, Joseph D. Tucker, Weiping Cai

**Affiliations:** Guangzhou Eighth People’s Hospital, Guangzhou, China; UNC-Project – China, Division of Infectious Diseases, Department of Medicine, UNC Chapel Hill School of Medicine, Chapel Hill, NC USA; Department of Medicine, Division of Gastroenterology Providence, Brown University School of Medicine, Rhode Island, USA; University of South Florida, Morsani College of Medicine, USF International, Tampa, FL USA; Division of Infectious Diseases, Department of Medicine, UNC Chapel Hill School of Medicine, Chapel Hill, NC USA

**Keywords:** Hepatitis C, Human immunodeficiency virus, HIV/HCV co-infection, Epidemiology, HCV genotype, Hepatic fibrosis

## Abstract

**Background:**

End-stage liver disease and hepatocellular carcinoma due to hepatitis C virus (HCV) co-infection are increasingly common causes of death among HIV-infected individuals. However, there are few clinical investigations of HIV/HCV co-infected individuals from low and middle-income nations. Here, we compare the epidemiology of HCV-infected and HIV/HCV co-infected individuals in Southern China and examine hepatic fibrosis scores in co-infected individuals.

**Methods:**

We conducted a retrospective cross-sectional study of treatment-naïve HIV/HCV co-infected and HCV mono-infected subjects. Bivariate and multivariate models were used to examine the association between demographics and HCV genotype. Among co-infected individuals, we also studied the relationship between fibrosis scores derived from non-invasive studies and HCV genotype.

**Results:**

Data were collected from 175 HCV-infected individuals, including 89 (51 %) HIV/HCV co-infected individuals. HIV/HCV co-infection was correlated with intravenous drug use (AOR 46.25, *p* < 0.001) and not completing high school (AOR 17.39, *p* < 0.001) in a multivariate model. HIV/HCV co-infected individuals were more likely to be infected with HCV genotype 6a (*p* < 0.0001) or 3a (*p* < 0.023), whereas increased fibrosis (FIB-4 score) was associated with HCV genotype 3a infection (β 2.18, *p* < 0.001).

**Discussion:**

Our results suggest that intravenous drug use is driving HIV/HCV co-infection in Southern China. While additional studies are needed, HCV genotype 6a is more common and genotype 3a appears to be associated with more severe hepatic fibrosis in co-infected individuals.

**Conclusions:**

Future HIV/HCV co-infection research in China should focus on at risk populations, HCV testing uptake, and genotype-specific treatment.

**Electronic supplementary material:**

The online version of this article (doi:10.1186/s12879-015-1135-1) contains supplementary material, which is available to authorized users.

## Background

Chronic infection with hepatitis C virus (HCV) has emerged as a significant contributor to morbidity and mortality in patients infected with human immunodeficiency virus (HIV) in the era of successful antiretroviral therapy (ART) implementation [1–3]. Driven by similar modes of transmission, in particular intravenous drug use, rates of dual infection over the years have risen and now comprise a significant portion of the HIV-infected population [4]. Total numbers of HIV-positive and HCV-positive individuals in China is estimated at 780,000 [5] and 8.9 million [6], respectively. In HIV patients currently on ART, approximately 18.2 % are co-infected with HCV [7]. Geographic features have shaped the HIV/HCV co-infection epidemic within China, with Southern China disproportionately affected due to its proximity to drug trafficking within the “Golden Triangle,” comprised of neighboring Myanmar, Laos, and Thailand [10]. Studies in Southern China have shown up to 60–95 % of people who inject drugs (PWID) are infected with HCV while co-infection with HIV ranges from 6–17 % [11–13].

The trend towards rising numbers of co-infected patients underscores the importance of studying the clinical and epidemiological interplay between HIV and chronic HCV infection. Studies have demonstrated that HIV co-infection accelerates HCV-associated liver fibrosis progression, a phenomenon possibly mediated by effects of HIV infection on fibrogenesis in the setting of immunosuppression [14, 15]. Consequently, the development of end-stage liver disease (ESLD) with cirrhosis and hepatocellular carcinoma occurs at a younger age in co-infected individuals [16]. With the normalization of HIV life expectancy with ART, liver disease is now the fastest growing cause of death among those who are co-infected [2, 3]. Treatment of either HIV [17] or HCV [18, 19] appears to reduce fibrosis progression and risk of ESLD. Recent advances in HCV therapeutics have simplified treatment regimens with sustained virologic response achieved in 84–94 % of HIV/HCV co-infected patients [20, 21]. For these reasons, treatment of HCV infection is clearly top priority in the care of co-infected persons. Despite therapeutic advances, obstacles in delivery of care in HIV/HCV co-infection remain with decreased treatment uptake secondary to a combination of active drug use, concerns regarding side effects, drug cost and availability, and lack of patient education [22, 23].

The potential morbidity and mortality associated with untreated HIV/HCV co-infection warrants a more complete epidemiological understanding in China, particularly in a region with higher prevalence such as Southern China. HCV genotype data is still essential for guiding therapeutic decisions and both laboratory and epidemiological prevalence data have been limited in low and middle income nations [24]. Genotype distribution and association with fibrosis scores in HIV-infected individuals will be vital to establishing optimal treatment regimens in China in the future. Here, we describe a cross-sectional study that documents that a high proportion of co-infected patients in Southern China are infected with HCV genotype 6a and suggests a significant correlation between HCV genotype 3a and severe fibrosis.

## Methods

### Study site

China’s third largest city, Guangzhou, has a population of over 8.5 million persons and is an economic hub within the center of the prosperous Pearl River Delta Region [25]. The city has a high prevalence of both HIV and HCV infection among key affected populations, including PWID [12]. The municipality of Guangzhou only has one public infectious disease hospital, the Guangzhou Eighth People’s Hospital. Its outpatient HIV clinic sees approximately 3000 HIV positive individuals monthly and administers free anti-retroviral (ART) medications through the national plan called Four Frees and One Care [26]. Although there is no analogous system for providing free of charge HCV therapy, pegylated interferon and ribavirin are available.

### Study population

All patients were enrolled from the Outpatient Hepatitis Clinic at Guangzhou Eighth People’s Hospital between 2008 and 2011. Patients from the HIV/HCV co-infected cohort were initially recruited as part of a large multi-center HCV treatment trial and this study is a secondary analysis of data from that trial. All participants at time of enrollment were older than 18 years of age with a positive IgG or IgM anti-HCV ELISA (Zhongshan Bioengineering, China) and detectable HCV RNA > 15 IU/ml (Roche Molecular Systems, USA). Participants were included if naïve to HCV treatment at time of enrollment. Among HIV/HCV co-infected individuals, all individuals had a positive HIV ELISA (Beijing Wantai, China) with a confirmatory Western blot (MP Biomedicals, Singapore). Due to contraindications to treatment, exclusion criteria included individuals with decompensated cirrhosis, severe cytopenias, pregnancy, breast-feeding status, renal failure, heart failure, or an AIDS-defining illness. A mono-infected cohort using the same criteria was recruited separately for the purpose of this study.

### Study procedure

Demographic and laboratory data were extracted retrospectively from hospital records for mono-infected individuals or at time of enrollment for co-infected individuals. Age, sex, ethnicity, education level, marital status, employment status, province of origin, year of HCV testing, and physician-reported HCV risk factors were recorded. As liver biopsy data were not routinely available and infrequently obtained at our clinic, non-invasive fibrosis indices were employed. Aspartate aminotransferase (AST), alanine aminotransferase (ALT), and platelet count were obtained from the same year as the initial HCV genotype testing and used to calculate APRI and FIB-4 fibrosis scores. The equation for APRI is as follows: (AST/upper limit of normal)/platelet count (expressed as platelets × 109/L) × 100 [27]. FIB-4 was calculated using the following formula: age [years] × AST [IU/L]/platelet count [expressed as platelets × 109/L] × (ALT1/2[IU/L]) [28]. Standardized APRI and FIB-4 cutoff values were used to classify fibrosis as “no significant fibrosis” (Class 1: APRI ≤0.5; FIB-4 ≤ 1.45), “intermediate status” (Class 2: APRI 0.51–1.5; FIB-4 1.46–3.25), or “significant fibrosis” (Class 3: APRI > 1.5; FIB-4 > 3.25) [27, 28].

HCV RNA was extracted from serum or plasma with the ViraRNA Mini Kit (Qiagen, USA). Amplified cDNA was produced using core protein (CP) and NS5B region-specific primers [29–31] (see Additional file 1). The cDNA amplimers were sequenced and HCV genotypes were confirmed using the Los Alamos HCV database. Sample HCV core gene sequences were aligned using CLUSTALW and a phylogenetic tree was created using a neighbor-joining method based on the alignment of CP sequences (Kimura 2-parameter model and bootstrap analysis with 1000 replicates), by Molecular Evolutionary Genetics Analysis software version 4.0 (MEGA 4.0).

### Data analysis

We examined correlates of HIV/HCV co-infection among all HCV-positive individuals enrolled in the study, and then examined fibrosis scores among HIV/HCV co-infected individuals. Demographic and laboratory differences between HCV mono-infected and HIV/HCV co-infected individuals were examined using the Pearson Chi squared test for categoric variables and the Student’s *t* test or Wilcoxon rank sum test in cases of non-normal distribution for continuous variables. A stepwise logistic bivariate regression analysis was used to investigate correlates of HIV/HCV co-infection among HCV-infected individuals. Variables associated with a *p*-value < 0.05 were included in the model. Bivariate and multivariate linear regression models with outcome of increased fibrosis score as a continuous variable were also examined. The Akaike Information Criterion (AIC) was used as a measure of relative-goodness-of-fit to discriminate among various estimated models. Our final set of models is based on a combination of AIC goodness-of-fit as well as inclusion of clinically relevant variables, for which we report the adjusted odds ratios (AOR). We used SPSS version 20 for Windows (Armonk, NY) [32], R 2.14 for Mac OS X (Vienna, Austria) [33], and Stata/IC 12.1 (College Station, TX) [34].

### Ethics statement

All individuals in this study provided written informed consent according to the principles of the Declaration of Helskini. The study was reviewed and considered exempt due to delinking of personal identifiers from clinical and laboratory data by the Guangzhou Eighth People’s Hospital Institutional Review Board (IRB # 57832455).

## Results

We examined data from 175 HCV-infected individuals, including 89 (51 %) HIV/HCV co-infected individuals. The baseline characteristics for study participants are listed in Table 1. The mean age of the entire cohort was 38.3 years and 69 % were male. There were significant demographic differences in gender, level of education, and route of HCV transmission. Clinically, median AST was higher in the co-infected group, although no differences were found in median ALT or mean platelet count. Both APRI score (*p* = 0.012) and FIB-4 score (*p* = 0.009) showed statistically significant differences between the two groups. Most individuals (96, 55 %) had no significant fibrosis based on FIB-4 score. Forty-nine (28 %) had intermediate fibrosis and 30 (17 %) had significant fibrosis. There was a more even distribution in APRI score with 68 (39 %) individuals with no fibrosis, 66 (38 %) with intermediate fibrosis, and 41 (23 %) with significant fibrosis.

**Table 1:** Demographic and clinical baseline characteristics

Demographic or clinical data	Mono-infection (*n* = 86)	Co-infection (*n* = 89)	Total (*n* = 175)	*p*-value
Age (y), mean ± SD	39.9 ± 12.6	36.6 ± 6.3	38.3 ± 9.9	0.133
Male gender, *n* (%)	49 (57)	72 (81)	121 (69)	**0.001**
Marital status, *n* (%)				0.609
Married	55 (64)	69 (78)	124 (71)	
Not married	13 (15)	20 (22)	33 (19)	
Unknown	18 (21)	0 (0)	18 (10)	
Education status, *n* (%)				**<0.001**
< HS education, *n* (%)	12 (14)	74 (83)	86 (49)	
> HS education	47 (55)	15 (17)	62 (35)	
Unknown	27 (31)	0 (0)	27 (15)	
Employment status, *n* (%)				0.280
Employed or student	47 (55)	56 (63)	103 (59)	
Unemployed	19 (22)	33 (37)	52 (30)	
Unknown	20 (23)	0 (0)	20 (11)	
HCV risk factor, *n* (%)				**<0.001**
IDU	3 (4)	72 (81)	75 (43)	
Blood transfusion	37 (43)	2 (2)	39 (22)	
Sexual	0 (0)	11 (12)	11 (6)	
Other^a^	46 (54)	4 (5)	50 (29)	
Unknown	25 (29)	1 (1)	26 (15)	
HCV genotype, *n* (%)				**<0.001**
1a	3 (4)	1 (1)	4 (2)	0.362
1b	57 (66)	15 (17)	72 (41)	**<0.001**
2a	5 (6)	1 (1)	6 (3)	0.113
3a	3 (3)	16 (18)	19 (11)	**0.002**
3b	6 (7)	9 (10)	15 (9)	0.459
6a	12 (14)	47 (53)	59 (34)	**<0.001**
AST, median (IQR)	38 (29–65)	54 (36–92)	43 (32–73)	**0.004**
ALT, median (IQR)	47.5 (32–90)	49 (33–81)	49 (32–85)	0.794
Platelet count (^10),	185 ± 69	179 ± 69	182 ± 69	0.644
mean ± SD				
APRI score^b^, *n* (%)				**0.012**
Class 1	43 (50)	26 (28)	68 (39)	
Class 2	26 (30)	40 (45)	66 (38)	
Class 3	17 (20)	24 (27)	41 (23)	
FIB-4 score^c^, *n* (%)				**0.009**
Class 1	55 (64)	41 (46)	96 (55)	
Class 2	15 (17)	34 (38)	49 (28)	
Class 3	16 (19)	14 (16)	30 (17)	

*Abbreviations:* *IDU* intravenous drug use

^a^Other iatrogenic is defined by reporting of HCV transmission route through other medical or dental routes besides blood transfusion

^b^APRI Categories: APRI Class 1 (Score < 0.5), APRI Class 2 (Score 0.51–1.5), APRI Class 3 (Score > 1.5)

^c^FIB-4 Categories: FIB-4 Class 1 (Score < 1.45), FIB-4 Class 2 (Score 1.46–3.25), FIB-4 Class 3 (Score > 3.25)

Bold *p*-values are significant at the 0.05 level

**Fig. 1:**
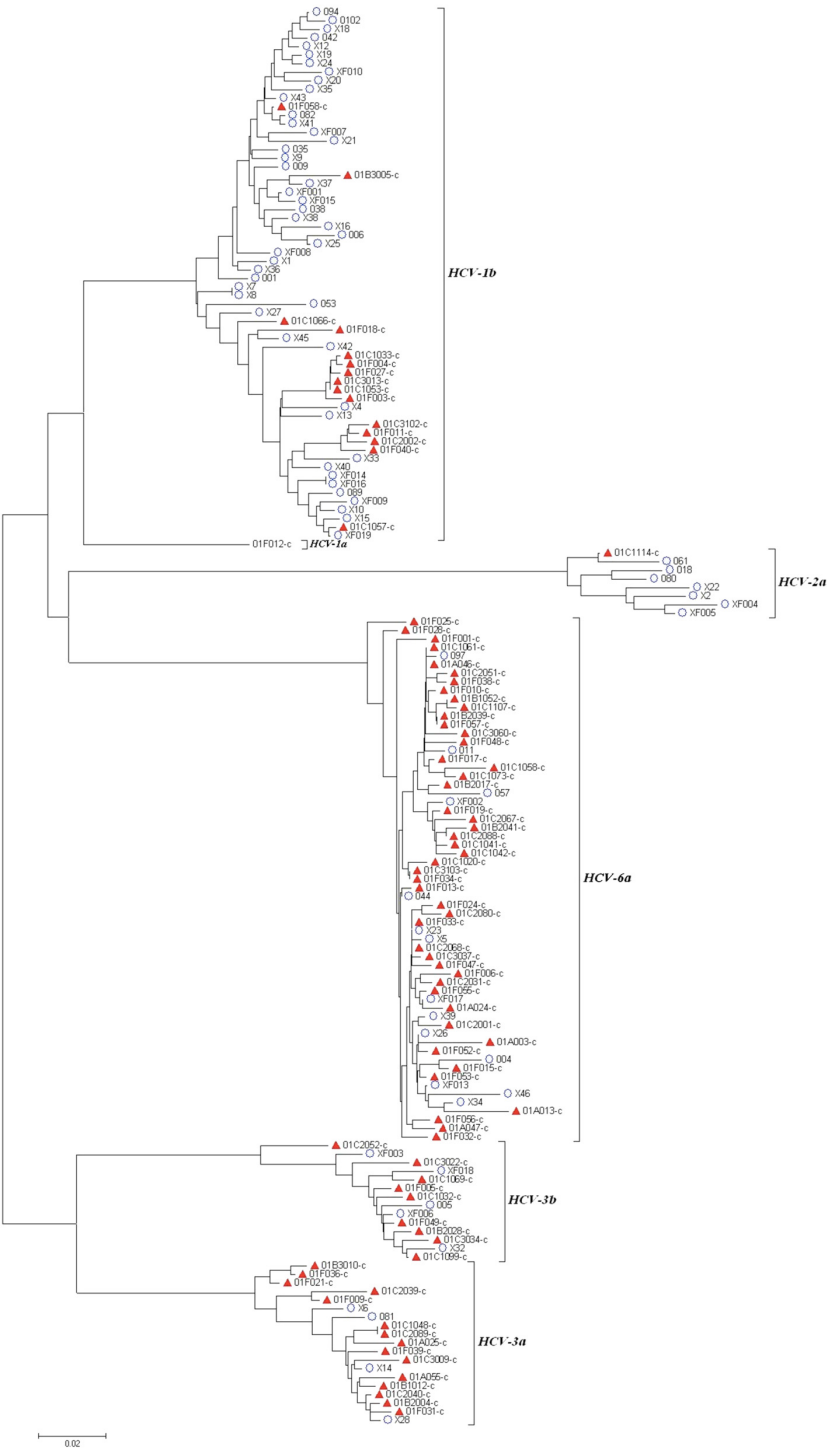
Phylogenetic tree analysis of HCV core protein gene sequences (*n* = 175). Shown are HCV mono-infected (blue circles) and HCV/HIV co-infected samples (red triangles)

Four HCV genotypes and six subtypes were found in our study population. The most common HCV genotype was 1b (41.1 %), followed by 6a (33.7 %), 3a (10.9 %), 3b (8.6 %), 2a (3.4 %), and 1a (2.3 %) as seen in the phylogenic tree (Fig. 1). Phylogenetic analysis revealed clustering of HIV/HCV co-infected individuals among genotype 1b. HCV genotype 2a was more common among HCV mono-infected individuals, while the large majority of those with genotype 6 and 3 infections were co-infected with HIV. These differences in genotype distribution were highly significant statistically in univariate analysis for genotype 1b (*p* <0.001), 3a (*p* = 0.002), and 6a (*p* < 0.001).

Bivariate analyses showed that HIV/HCV co-infected individuals were more likely to report a history of intravenous drug use (*p* <0.001), have less than a high school education (*p* < 0.001), be male (*p* <0.001) and younger (*p* <0.031) compared to HCV mono-infected individuals (Table 2). HIV/HCV co-infected individuals were more likely to have HCV genotype 6a (*p* < 0.0001) or 3a (*p* < 0.023). Multivariate analyses showed that HIV/HCV co-infection was correlated with intravenous drug use history (*p* < 0.001), not completing high school (*p* < 0.001), and less likely to have a blood transfusion history (*p* < 0.005).

**Table 2:** Correlates of HIV/HCV co-infection using bivariate (OR) and multivariate logistic regression (AOR) modeling

Characteristic	OR (95 % CI)	*p*-value	AOR (95 % CI)	*p*-value
IDU transmission	117.18 (33.00–416.13)	**<0.0001**	46.25 (8.39–254.88)	**<0.001**
Did not complete high school	19.32 (8.32–44.87)	**<0.0001**	17.39 (4.45–67.96)	**<0.001**
Blood transmission	0.03 (0.01–0.13)	**<0.0001**	0.08 (0.01–0.57)	**0.005**
HCV genotype 6a	6.02 (2.97–12.22)	**<0.0001**		
HCV genotype 3a	3.83 (1.21–12.14)	**0.023**		
Male	3.20 (1.62–6.31)	**0.001**		
Guangdong province	3.07 (1.27–7.41)	**0.013**		
Increasing age	0.97 (0.94–0.997)	**0.031**		
Unknown HCV risk factor	0.18 (0.05–0.65)	**0.009**		
HCV genotype 1b	0.13 (0.07–0.26)	**<0.0001**	Model AIC =72.14	

*Abbreviations:* *OR* odds ratio, *AOR* adjusted odds ratio, *CI* confidence interval, *IDU* intravenous drug use, *AIC* Aikaike information criteria. Missing data were excluded from statistical analysis. Bold *p*-values are significant at the 0.05 level

Among HIV/HCV co-infected individuals (*n* = 89), more advanced hepatic fibrosis according to the FIB-4 score was associated with HCV genotype 3a infection (Table 3). Increasing FIB-4 score was also associated with age (*p* = 0.004) and unemployment (*p* = 0.010). Presence of ART use and CD4 count did not correlate strongly with level of fibrosis. Associations with APRI score were much weaker in our model and overall there were no significant findings.

**Table 3:** Associations with FIB-4 score among HIV/HCV co-infected individuals (*n* = 89)

Correlate			β Coefficient (95 % CI)	*p*-value	β Adjusted Coefficient (95 % CI)	*p*-value
Age, years			0.10 (0.03,0.17)	**0.005**	0.09 (0.030, 0.153)	**0.004**
Unemployed			1.24 (0.36,2.13)	**0.006**	1.07 (0.26, 1.87)	**0.010**
HCV Genotype 3a			2.15 (1.01,3.28)	**<0.001**	2.18 (1.17, 3.18)	**<0.001**
Immunosuppression	ART Naïve (*n* = 52)	ART (*n* = 37)				
CD4^+^ ≤ 50 cells/μL	15	0	2.00 (0.02,3.98)	**0.048**	2.39 (0.73, 4.07)	**0.005**
CD4^+^ 51–200 cells/μL	24	3	1.07 (−0.79,2.92)	0.256	1.30 (−0.25, 2.84)	0.098
CD4^+^ 201–350 cells/μL	7	20	1.42 (−0.44,3.27)	0.132	1.65 (0.10, 3.20)	**0.037**
CD4^+^ 351–500 cells/μL	5	9	0.27 (−1.74,2.27)	0.793	0.44 (−1.23, 2.11)	0.600
CD4^+^ 501–1000 cells/μL	1	5	Referent		Referent	

*Abbreviations:* *ART* antiretroviral therapy, *CI* confidence interval. For HCV mono-infected patients, HCV genotype 3a was not found to correlate with increased FIB-4 score (*p* = 0.914). No significant correlations were found with APRI score. Bold *p*-values are significant at the 0.05 level

## Discussion

The results from our cross-sectional study suggest that intravenous drug use is the main driver of HIV/HCV co-infection in Guangzhou. This finding is consistent with both Chinese [11, 12] and global literature [8, 9] on HIV/HCV co-infection. The ongoing risk of transmission of HIV in HCV positive PWID highlights the health systems gap between HIV and HCV service infrastructures [35]. HCV mono-infected individuals in China do not have access to the same education and preventative services as HIV-infected individuals, leading to continued high-risk behaviors and acquisition of co-infection. While China’s needle exchange programs [36, 37] and methadone maintenance programs [38, 39] have achieved success, HIV and HCV testing delays are still common at methadone maintenance programs [40, 41]. The structure of methadone maintenance programs in China, however, is a strong foundation on which to build testing of high-risk drug users, link them to care, and initiate therapy.

Several conclusions can be drawn from the distribution of HCV genotypes we found in our two cohorts. First, the clustering of HCV genotype 1b within the HIV/HCV co-infected cohort suggests a common HCV transmission source in Southern China. Furthermore, the phylogenetic clustering of co-infection implies HIV transmission is common in a subgroup of patients with HCV genotype 1b. The most frequently represented genotype in prior studies on both HCV mono-infected individuals and provinces outside of Guangdong is genotype 1b [30, 42]. Genotype 1b was also the dominant genotype in our HCV mono-infected cohort, representing 64 % of the cohort. In comparison, over half of the co-infected cohort consisted of genotype 6a. The differential distribution of genotypes in mono- and co-infection is likely a product of differing modes of disease transmission, with blood transfusions the more common route in mono-infection. Our study corroborates prior findings of increased rates of genotype 6a among co-infected patients within Southern China [30, 43, 44], specifically the Guangdong province. The increased prevalence of genotype 6a in co-infection is primarily driven by an epidemic of injection drug use in Southern China. Epidemiological studies in Southeast Asia have uncovered high rates of genotype 6a in mono-infection with injection drug use as a principal risk factor [45]. The geographic proximity to Southeast Asia and presence of drug trafficking and use likely explains route of entry of genotype 6a into the Guangdong province. The persistence of genotype 6a in co-infection is concerning for ongoing transmission potential in PWID and highlights the importance of targeting this at-risk population. Patients with genotype 6a have responded well to peg-interferon and ribavirin with higher SVR rates and lower relapse rates compared to other genotypes [46]. In vitro studies on efficacy of novel protease inhibitors (PI) on genotype 6a have also demonstrated good response [47], and a limited but promising U.S.-based phase 2 clinical trial on genotype 6 patients demonstrated all five HCV genotype 6 patients enrolled achieving SVR on 24 weeks of sofosbuvir in combination with peg-interferon and ribavirin [48]. Extending such trials in Southern China in which there is a high prevalence of genotype 6 would be recommended.

We also identified correlates of fibrosis severity in the HIV/HCV co-infected population, as fibrosis is an important predictor of response to therapy [49]. The most notable finding was an association between genotype 3a and prevalence of advanced hepatic fibrosis (*p*-value < 0.001) by FIB-4, although not by APRI. Overall, FIB-4 and APRI scores did not show strong correlations in our analysis and may reflect our exclusion of patients with decompensated cirrhosis or severe cytopenias. The lack of concordance in FIB-4 and APRI results is also worth noting, given prior studies showing only low to moderate concordance, especially in patients with HIV [50]. Infection with HCV genotype 3, which was found in 19.5 % of our study cohort, has been established as an independent risk factor for accelerated fibrosis progression in HCV mono-infection [51]. The mechanism behind this observation is thought to be presence of concomitant hepatic steatosis with genotype 3 as a result of its specific genetic and molecular composition and influence on lipid metabolism [52]. Potentially a consequence of more severe hepatic fibrosis is a clear inferiority in treatment response attained with traditional HCV treatment regimens on genotype 3 [51]. Labeled as difficult to treat, genotype 3a has also shown the most in vitro resistance to newer generation PIs [53]. A clinical trial with sofosbuvir has demonstrated moderate treatment success with genotype 3 [54], at generally lower SVR rates than other genotypes. Further studies are needed on the effect of genotype 3 induced hepatic steatosis on fibrosis and its potential interaction with therapy.

Several limitations of this study should be noted. First, our sample size is small compared to the scale of HIV/HCV co-infection in China. All participants came from a single clinic in Guangzhou and co-infected patients were enrolled in an interventional study; as such, participants in this study may not be representative of HCV-infected individuals from the region. However, the HCV/HIV co-infection genotypes we identified in our cohort are consistent with literature; moreover, the subjects we studied came from a broad, province-wide catchment area. Second, there may be significant selection bias present in our analysis due to 1) missing demographic data from the mono-infected cohort and 2) lack of matching between the mono-infected and co-infected cohorts. We were also not able to collect information on alcohol use, herbal use, duration of HCV infection, insulin resistance, HIV viral load, and use of hepatotoxic drugs including ART dosing and duration. Despite the bias introduced, we believe our findings demonstrating a strong association between HIV/HCV co-infection with IDU and lower education status is valid given similar findings in previously published epidemiological research, particularly in China [55, 56]. Lastly, we did not obtain liver biopsies or transient elastography. At the same time, APRI and FIB-4 scores demonstrate high specificity for Class 1 and Class 3 fibrosis [57]. In co-infection, APRI and FIB-4 have been noted to accurately predict fibrosis with a high negative predictive value for ruling out advanced fibrosis but with a poor positive predictive value [58]. Non-invasive indices of staging will prove to be invaluable tools in regions where biopsies or Fibroscan are not readily available. Their use will likely expand even in high-income areas as we move towards less invasive and more cost-effective care.

## Conclusions

China has an estimated 38 million HCV-infected individuals [59]. HCV is an emerging public health concern and understanding correlates of transmission and progression of HIV/HCV co-infection will be crucial as other HIV-related complications decrease and complications of liver disease rise both nationally and worldwide. The epidemiological findings in our study impact current HCV treatment, as currently available direct-acting agent (DAA) regimens are genotype specific. More studies are needed to determine optimal treatment strategies and provide appropriate guidance on the highly complex treatment algorithms anticipated for HCV. While China has already made great strides in treatment of HIV by increasing ART uptake, our data encourage further research and the development of health policies focused on PWID, HCV testing uptake, and HCV genotype-specific treatment in Southern China.

## Availability of supporting data

Phylogenetic tree data available from the Dryad Digital Repository: http://dx.doi.org/10.5061/dryad.6mt70.

## Additional file

Additional file 1:
**HCV genotyping primers for NS5B and core protein regions.** (DOCX 62 kb)

## References

[1] Ly KN, Xing J, Klevens RM, Jiles RB, Ward JW, Holmberg SD (2012). The increasing burden of mortality from viral hepatitis in the United States between 1999 and 2007. Ann Intern Med.

[2] Monga HK, Rodriguez-Barradas MC, Breaux K, Khattak K, Troisi CL, Velez M, Yoffe B (2001). Hepatitis C virus infection-related morbidity and mortality among patients with human immunodeficiency virus infection. Clin Infect Dis.

[3] Weber R, Sabin C, Friis-Møller N, for the The Data Collection on Adverse Events of Anti-HIV Drugs Study Group (2006). Liver-related deaths in persons infected with the human immunodeficiency virus: The D:A:D study. Arch Intern Med.

[4] Taylor LE, Swan T, Mayer KH (2012). HIV coinfection with hepatitis C virus: evolving epidemiology and treatment paradigms. Clin Infect Dis.

[5] China MoHotPsRo. 2012 China AIDS response progress report. In. Edited by China MoHotPsRo. China; 2012.

[6] Gower E, Estes C, Blach S, Razavi-Shearer K, Razavi H (2014). Global epidemiology and genotype distribution of the hepatitis C virus infection. J Hepatol.

[7] Zhang F, Zhu H, Wu Y, Dou Z, Zhang Y, Kleinman N, Bulterys M, Wu Z, Ma Y, Zhao D (2014). HIV, hepatitis B virus, and hepatitis C virus co-infection in patients in the China National Free Antiretroviral Treatment Program, 2010–12: a retrospective observational cohort study. Lancet Infect Dis.

[8] Mathers BM, Degenhardt L, Phillips B, Wiessing L, Hickman M, Strathdee SA, Wodak A, Panda S, Tyndall M, Toufik A (2008). Global epidemiology of injecting drug use and HIV among people who inject drugs: a systematic review. Lancet.

[9] Nelson PK, Mathers BM, Cowie B, Hagan H, Des Jarlais D, Horyniak D, Degenhardt L (2011). Global epidemiology of hepatitis B and hepatitis C in people who inject drugs: results of systematic reviews. Lancet.

[10] Beyrer C, Razak MH, Lisam K, Chen J, Lui W, Yu XF (2000). Overland heroin trafficking routes and HIV-1 spread in south and south-east Asia. Aids.

[11] Garten RJ, Zhang J, Lai S, Liu W, Chen J, Yu XF (2005). Coinfection with HIV and hepatitis C virus among injection drug users in southern China. Clin Infect Dis.

[12] Bao YP, Liu ZM (2009). Systematic review of HIV and HCV infection among drug users in China. Int J STD AIDS.

[13] Shen YZ, Wang ZY, Qi TK, Jiang XY, Song W, Tang Y, Wang JR, Liu L, Zhang RF, Zheng YF (2013). Serological survey of viral hepatitis markers among newly diagnosed patients with HIV/AIDS in China. HIV Med.

[14] Macias J, Berenguer J, Japon MA, Giron JA, Rivero A, Lopez-Cortes LF, Moreno A, Gonzalez-Serrano M, Iribarren JA, Ortega E (2009). Fast fibrosis progression between repeated liver biopsies in patients coinfected with human immunodeficiency virus/hepatitis C virus. Hepatology.

[15] Reiberger T, Ferlitsch A, Sieghart W, Kreil A, Breitenecker F, Rieger A, Schmied B, Gangl A, Peck-Radosavljevic M (2010). HIV-HCV co-infected patients with low CD4+ cell nadirs are at risk for faster fibrosis progression and portal hypertension. J Viral Hepat.

[16] Mohsen AH, Easterbrook P, Taylor CB, Norris S (2002). Hepatitis C and HIV-1 coinfection. Gut.

[17] Mehta SH, Thomas DL, Torbenson M, Brinkley S, Mirel L, Chaisson RE, Moore RD, Sulkowski MS (2005). The effect of antiretroviral therapy on liver disease among adults with HIV and hepatitis C coinfection. Hepatology.

[18] Berenguer J, Zamora FX, Carrero A, Von Wichmann MA, Crespo M, Lopez-Aldeguer J, Aldamiz-Echevarria T, Montes M, Quereda C, Tellez MJ (2014). Effects of sustained viral response in patients with HIV and chronic hepatitis C and nonadvanced liver fibrosis. J Acquir Immune Defic Syndr.

[19] Soriano V, Labarga P, Ruiz-Sancho A, Garcia-Samaniego J, Barreiro P (2006). Regression of liver fibrosis in hepatitis C virus/HIV-co-infected patients after treatment with pegylated interferon plus ribavirin. Aids.

[20] Molina J-M, Orkin C, Iser DM, Zamora F-X, Nelson M, Stephan C, Massetto B, Gaggar A, Ni L, Svarovskaia E (2015). Sofosbuvir plus ribavirin for treatment of hepatitis C virus in patients co-infected with HIV (PHOTON-2): a multicentre, open-label, non-randomised, phase 3 study. Lancet.

[21] Sulkowski MS, Eron JJ, Wyles D, Trinh R, Lalezari J, Wang C, Slim J, Bhatti L, Gathe J, Ruane PJ (2015). Ombitasvir, paritaprevir co-dosed with ritonavir, dasabuvir, and ribavirin for hepatitis C in patients co-infected with HIV-1: a randomized trial. JAMA.

[22] Grebely J, Genoway KA, Raffa JD, Dhadwal G, Rajan T, Showler G, Kalousek K, Duncan F, Tyndall MW, Fraser C (2008). Barriers associated with the treatment of hepatitis C virus infection among illicit drug users. Drug Alcohol Depend.

[23] Reiberger T, Obermeier M, Payer BA, Baumgarten A, Weitner L, Moll A, Christensen S, Koppe S, Kundi M, Rieger A (2011). Considerable under-treatment of chronic HCV infection in HIV patients despite acceptable sustained virological response rates in a real-life setting. Antivir Ther.

[24] Averhoff FM, Glass N, Holtzman D (2012). Global burden of hepatitis C: considerations for healthcare providers in the United States. Clin Infect Dis.

[25] United Nations. Population distribution, urbanization, internal migration and development: an international perspective. In. Edited by Department of Economic and Social Affairs PD. New York, NY; 2011.

[26] Zhang F, Haberer JE, Wang Y, Zhao Y, Ma Y, Zhao D, Yu L, Goosby EP (2007). The Chinese free antiretroviral treatment program: challenges and responses. Aids.

[27] Wai C, Greenson J, Fontana R, Kalbfleisch J, Marrero J, Conjeevaram H, Lok A (2003). A simple noninvasive index can predict both significant fibrosis and cirrhosis in patients with chronic hepatitis C. Hepatology.

[28] Vallet-Pichard A, Malet V, Nalpas B, Verkarre V, Nalpas A (2007). FIB-4: an inexpensive and accurate marker of fibrosis in HCV infection. Comparison with liver biopsy and FibroTest. Hepatology.

[29] Lole KS, Jha JA, Shrotri SP, Tandon BN, Prasad VG, Arankalle VA (2003). Comparison of hepatitis C virus genotyping by 5′ noncoding region- and core-based reverse transcriptase PCR assay with sequencing and use of the assay for determining subtype distribution in India. J Clin Microbiol.

[30] Lu L, Nakano T, He Y, Fu Y, Hagedorn CH, Robertson BH (2005). Hepatitis C virus genotype distribution in China: predominance of closely related subtype 1b isolates and existence of new genotype 6 variants. J Med Virol.

[31] Enomoto N, Takada A, Nakao T, Date T (1990). There are two major types of hepatitis C virus in Japan. Biochem Biophys Res Commun.

[32] IBM Corporation. IBM SPSS statistics for windows. In*.*, 20.0 edn. Armonk, NY (USA); 2011.

[33] R Development Core Team. R: a language and environment for statistical computing. In*.* Edited by R Foundation for Statistical Computing. Vienna, Austria; 2003.

[34] StataCorp. Stata statistical software. In*.*, vol. 12. College Station, TX: StataCorp LP; 2011.

[35] Grebely J, Oser M, Taylor LE, Dore GJ (2013). Breaking down the barriers to hepatitis C virus (HCV) treatment among individuals with HCV/HIV coinfection: action required at the system, provider, and patient levels. J Infect Dis.

[36] Hammett T, Des Jarlais D, Kling R, Kieu B, McNicholl J, Wasinrapee P, McDougal J, Liu W, Chen Y, Meng D (2012). Controlling HIV epidemics among injection drug users: eight years of Cross-Border HIV prevention interventions in Vietnam and China. PLoS One.

[37] Wu ZY, Luo W, Sullivan SG, Rou K, Lin P, Liu W (2007). Evaluation of a needle social marketing strategy to control HIV among injecting drug users in China. AIDS.

[38] Lin CQ, Wu ZY, Rou KM, Yin WY, Wang CH, Shoptaw S (2010). Structural-level factors affecting implementation of the methadone maintenance therapy program in China. J Subst Abuse Treat.

[39] Yin WY, Hao Y, Sun XH, Gong XL, Li F, Li J (2010). Scaling up the national methadone maintenance treatment program in China: achievements and challenges. Int J Epidemiol.

[40] Zhang L, Chow EP, Zhuang X, Liang Y, Wang Y, Tang C, Ling L, Tucker JD, Wilson DP (2013). Methadone maintenance treatment participant retention and behavioural effectiveness in china: a systematic review and meta-analysis. PLoS One.

[41] Xia Y-H, McLaughlin MM, Chen W, Ling L, Tucker JD (2013). HIV and hepatitis C virus testing delays at methadone clinics in Guangdong province, china. PLoS One.

[42] Chen WL, Nie JM, Cai WP, Yuan XZ, Hu FY, Wei SJ, Tang YB, Zhang FC, Tang XP (2011). Analysis of hepatitis C virus (HCV) subtypes in HIV/HCV co-infected and HCV mono-infected individuals in Guangdong province. Zhonghua Gan Zang Bing Za Zhi.

[43] Fu Y, Wang Y, Xia W, Pybus OG, Qin W, Lu L, Nelson K (2011). New trends of HCV infection in China revealed by genetic analysis of viral sequences determined from first-time volunteer blood donors. J Viral Hepat.

[44] Rong X, Xu R, Xiong H, Wang M, Huang K, Chen Q, et al. Increased prevalence of hepatitis C virus subtype 6a in China: a comparison between 2004–2007 and 2008–2011. Arch Virol. 2014;159(12):3231-7. 10.1007/s00705-014-2185-1 PMC4221604 25085624

[45] Chao DT, Abe K, Nguyen MH (2011). Systematic review: epidemiology of hepatitis C genotype 6 and its management. Aliment Pharmacol Ther.

[46] Zhou YQ, Wang XH, Hong GH, Zhu Y, Zhang XQ, Hu YJ, Mao Q (2011). Twenty-four weeks of pegylated interferon plus ribavirin effectively treat patients with HCV genotype 6a. J Viral Hepat.

[47] Gottwein JM, Scheel TK, Jensen TB, Ghanem L, Bukh J (2011). Differential efficacy of protease inhibitors against HCV genotypes 2a, 3a, 5a, and 6a NS3/4A protease recombinant viruses. Gastroenterology.

[48] Kowdley KV, Lawitz E, Crespo I, Hassanein T, Davis MN, DeMicco M, Bernstein DE, Afdhal N, Vierling JM, Gordon SC (2013). Sofosbuvir with pegylated interferon alfa-2a and ribavirin for treatment-naive patients with hepatitis C genotype-1 infection (ATOMIC): an open-label, randomised, multicentre phase 2 trial. Lancet.

[49] Corchado S, Lopez-Cortes LF, Rivero-Juarez A, Torres-Cornejo A, Rivero A, Marquez-Coello M, Giron-Gonzalez JA (2014). Liver fibrosis, host genetic and hepatitis C virus related parameters as predictive factors of response to therapy against hepatitis C virus in HIV/HCV coinfected patients. PLoS One.

[50] Mendeni M, Foca E, Gotti D, Ladisa N, Angarano G, Albini L, Castelnuovo F, Carosi G, Quiros-Roldan E, Torti C (2011). Evaluation of liver fibrosis: concordance analysis between noninvasive scores (APRI and FIB-4) evolution and predictors in a cohort of HIV-infected patients without hepatitis C and B infection. Clin Infect Dis.

[51] Tapper EB, Afdhal NH (2013). Is 3 the new 1: perspectives on virology, natural history and treatment for hepatitis C genotype 3. J Viral Hepat.

[52] Gordon A, McLean CA, Pedersen JS, Bailey MJ, Roberts SK (2005). Hepatic steatosis in chronic hepatitis B and C: predictors, distribution and effect on fibrosis. J Hepatol.

[53] Li Y-P, Ramirez S, Humes D, Jensen SB, Gottwein JM, Bukh J (2014). Differential Sensitivity of 5’UTR-NS5A Recombinants of Hepatitis C Virus Genotypes 1–6 to Protease and NS5A Inhibitors. Gastroenterology.

[54] Jacobson IM, Gordon SC, Kowdley KV, Yoshida EM, Rodriguez-Torres M, Sulkowski MS, Shiffman ML, Lawitz E, Everson G, Bennett M (2013). Sofosbuvir for hepatitis C genotype 2 or 3 in patients without treatment options. N Engl J Med.

[55] Garten RJ, Lai S, Zhang J, Liu W, Chen J, Vlahov D, Yu XF (2004). Rapid transmission of hepatitis C virus among young injecting heroin users in Southern China. Int J Epidemiol.

[56] Dong C, Huang ZJ, Martin MC, Huang J, Liu H, Deng B, Lai W, Liu L, Yang Y, Hu Y (2014). The impact of social factors on human immunodeficiency virus and hepatitis C virus co-infection in a minority region of Si-chuan, the People’s Republic of China: a population-based survey and testing study. PLoS One.

[57] Bedossa P, Dargere D, Paradis V (2003). Sampling variability of liver fibrosis in chronic hepatitis C. Hepatology.

[58] Shah A, Smith P, Sterling R (2011). Comparison of FIB-4 and APRI in HIV-HCV coinfected patients with normal and elevated ALT. Dig Dis Sci.

[59] Lavanchy D (2009). The global burden of hepatitis C. Liver Int.

