# Supplementary material for: Genotypic distribution and hepatic fibrosis among HIV/HCV co-infected individuals in Southern China: a retrospective cross-sectional study
Source: BMC Infect Dis. 2015 Sep 30;15:401. doi: 10.1186/s12879-015-1135-1 (PMC4589973; doi:10.1186/s12879-015-1135-1)
Supplement: Additional file 1: — HCV genotyping primers for NS5B and core protein regions. (DOCX 62 kb) [file 12879_2015_1135_MOESM1_ESM.docx]

**Table S1. HCV Genotyping Primers for NS5B and Core Protein Regions.**

| **Core Protein Primers** | | |
| --- | --- | --- |
| Outside Primers | Sense | Position |
| 5’-ACTGCCTGATAGGGTGCTTGC-3’ | + | 288 |
| 5’-ATGTACCCCATGAGGTCGGC-3’ | - | 732 |
| Inside Primers |  |  |
| 5’-AGGTCTCGTAGACCGTGCA-3’ | + | 321 |
| 5’-CATGTGAGGGTATCGATGAC-3’ | - | 705 |
| **NS5B Primers** | | |
| Outside Primers | Sense | Base Pairs |
| 5’-TGGGSTTYTCSTATGAYACCMGBTGYTTTGA-3’ | + | 8245 |
| 5’-ARTACCTRGTCATAGCCTCCGTGAA-3’ | - | 8616 |
| Inside Primers |  |  |
| 5’-TATGAYACCCGCTGYTTTGACTCCAC-3’ | + | 8256 |
| 5’-GTCATAGCCTCCGTGAAGGCTC-3’ | - | 8611 |
